# Understanding manufacturing repurposing: a multiple-case study of ad hoc healthcare product production during COVID-19

**DOI:** 10.1007/s12063-022-00297-1

**Published:** 2022-07-28

**Authors:** Wan Ri Ho, Omid Maghazei, Torbjørn H. Netland

**Affiliations:** Department of Management, Technology and Economics, ETH Zurich, Zurich, Switzerland

**Keywords:** Manufacturing repurposing, COVID-19, Causal loop diagram, Open innovation, Manufacturing capabilities, Corporate philanthropy

## Abstract

The repurposing of manufacturing facilities has provided a solution to the surge in demand for healthcare products during the COVID-19 pandemic. Despite being a widespread and important phenomenon, manufacturing repurposing has received scarce research. This paper develops a grounded understanding of the key factors that influence manufacturing repurposing at the macro and micro levels. We collected rich qualitative data from 45 case studies of firms’ repurposing initiatives during COVID-19. Our study focuses on four types of healthcare products that experienced skyrocketing demand during the first months of the COVID-19 pandemic: face shields, facemasks, hand sanitizers, and medical ventilators. Based on the case studies, we identify and generalize driving factors for manufacturing repurposing and their relationships, which are summarized in causal loop diagrams at both macro and micro levels. Our research provides practitioners, policymakers, and scholars with a conceptual understanding of the phenomenon of manufacturing repurposing. It helps manufacturing managers understand why, when, and how they should engage in manufacturing repurposing and informs policymakers when and how to tailor incentive policies and support schemes to changing situations. Scholars can build on our work to develop and test dynamic system–behavior models of the phenomenon or to pursue other research paths we discover. The world stands to benefit from improved manufacturing repurposing capabilities to be better prepared for future disruptions.

## Introduction

As the COVID-19 pandemic swept across the world in 2020, the demand for particular healthcare equipment skyrocketed far beyond the level of any safety stock (Hald and Coslugeanu 2021). Manufacturing repurposing has been considered a rapid response to addressing the global shortage of critical items during the COVID-19 pandemic (Joglekar et al. 2020; López-Gómez et al. 2020). Manufacturers from different industries engaged in manufacturing repurposing either to gain goodwill or to capture the business opportunities presented (Betti and Heinzmann 2020; López-Gómez et al. 2020). Firms have particularly started to produce personal protective equipment (PPE) or medical equipment products. For example, beer manufacturer BrewDog began producing hand sanitizers, sports car manufacturer Ferrari manufactured respirator valves, and luxury label Prada made facemasks (Netland 2020; Garza-Reyes et al. 2021). In other cases, firms sought to fight the pandemic by inventing new products (e.g., modified scuba masks for ventilators and hygienic surgical gowns) or finding new ways to use novel technologies (e.g., collaborative robots and additive manufacturing) (Malik et al. 2020). However, these efforts have raised substantial challenges and risks (Garza-Reyes et al. 2021). For example, there has been high uncertainty related to the dynamics of the pandemic, and firms have generally lacked experience in repurposing manufacturing. During the past two years, the scale and scope of manufacturing repurposing have been unprecedented, which raises many intriguing questions for research.

We define manufacturing repurposing as a firm’s rapid conversion of capacities and capabilities to produce new-to-the-firm products. Manufacturing repurposing has been a strong phenomenon in the industry during COVID-19, but it is almost entirely new to the literature. In particular, the literature on manufacturing repurposing in the context of pandemics is in its infancy (Garza-Reyes et al. 2021). Existing reports have barely started to explain why and how manufacturing firms repurposed to respond to COVID-19 (e.g., Ashforth 2020; Avery 2020; De Massis and Rondi 2020; George et al. 2020; Lawton et al. 2020; Shepherd 2020; Rouleau et al. 2021). In contrast, there is already extensive literature on the effects of COVID-19 on existing operations and supply chains (Phillips et al. 2022; Barbieri et al. 2020; Naz et al. 2021; Reed 2021; Yu et al. 2021). Therefore, there should be ample opportunity to contribute new insights into manufacturing repurposing, considering the extent and variety of repurposing during the COVID-19 pandemic. In this paper, we ask the following macro-and micro-level research questions: What factors affect manufacturing repurposing activities, and how do they relate to each other?

Addressing our research question, we contribute an understanding of manufacturing repurposing and the factors that affect its dynamic development. As one of the first multi-case empirical analyses of manufacturing repurposing, this is a novel contribution to the emerging literature. We use a multiple case study approach to map and analyze a large number of manufacturing repurposing initiatives during the COVID-19 pandemic. We focus on healthcare products that experienced explosive demand growth during spring 2020, more precisely from March to June 2020, depending on location. After analyzing the data via structured coding methods, we visualized the system dynamics of manufacturing repurposing in causal loop diagrams at the macro and micro levels. By bringing forward the key constructs of manufacturing repurposing, we lay a foundation for future research. The causal loop diagrams also provide practical insights for practitioners and policymakers, which can help improve decision-making processes in future emergencies. We also elaborate on the challenges and opportunities of manufacturing repurposing and outline promising research avenues.

The remainder of this paper is structured as follows. Section 2 provides a literature review of manufacturing repurposing. Section 3 details the research methodology. Section 4 presents our structured qualitative analysis. Section 5 summarizes the results in the form of macro- and micro-level causal loop diagrams. Section 6 discusses the implications of this study for both research and practice as well as its limitations and outlook. Section 7 concludes the paper.

## Literature review

Manufacturing repurposing is not a new phenomenon, but it lacks a dedicated and established stream of research. During all kinds of crises throughout history, humans have ingeniously developed ways to produce the needed products and tools to fix arising problems. During wartime, for example, repurposing production capacities to produce armory, ammunition, and other products in high demand is normal (e.g., Overy 1994). During natural disasters or other emergency events, local needs often require swift responses from local companies, which can help by producing products other than they normally do. For example, during the Aisin Seiki fire in the Kariya factory in 1997, Toyota’s supply of brake fluid valves was disrupted. This disruption drove Toyota to request other suppliers to repurpose production lines to produce valves for Toyota; within a short week, several companies began producing the needed valves. Manufacturing history is ripe with such stories, but they have not been studied collectively as phenomena. However, this is changing due to the unprecedented scale and scope of manufacturing repurposing the world has experienced during the COVID-19 pandemic.

During the recent pandemic, “manufacturing/production repurposing” has been used as a term to represent activities where a manufacturer uses its current capacities and capabilities to shift production to high-demand healthcare products like ventilators, facemasks, or sanitizers (e.g., Betti and Heinzmann 2020; López-Gómez et al. 2020). Scholars have picked up this term and studied the phenomenon using a variety of problem statements and approaches. The three dominant streams in the nascent literature have been: (1) barriers and success factors for successful repurposing, (2) supply chain issues, and (3) innovation.

Regarding the first stream of literature, Okorie et al. (2020) evaluate manufacturing repurposing as a firm-level pandemic response tool and identify enablers and barriers to repurposing. In particular, Okorie et al. (2020) recommend that manufacturing companies increase their flexibility, accelerate the adoption of digital technologies, and improve organizational processes such as decision making and organizational learning during pandemic and post-pandemic situations. The role of digital transformation in swift repurposing has also been emphasized by Soldatos et al. (2021). Relatedly, Poduval et al. (2021) use a model-based approach to identify and rank 11 types of barriers that played a central role in the repurposing of an existing manufacturing plant. Poduval et al. (2021) show that the identified barriers are interrelated and highlight the complexity of the manufacturing repurposing phenomenon. However, none of these studies have aimed to provide an understanding of all the internal and external factors that affect manufacturing repurposing and their causal relationships.

The second stream takes a supply chain perspective on manufacturing repurposing. For example, Falcone et al. (2022) use the concept of supply chain plasticity, which is defined as a firm’s “capability of rapidly making major changes to a supply chain to accommodate significant shifts in the business environment” (Zinn and Goldsby 2019, p. 184). Falcone et al. (2022) argue that the more supply chain plasticity is developed in a firm, the more capability the firm has to repurpose existing operations during disruptions. Some industrial reports also extrapolated the repurposing concept to supply chains, which could increase resilience and social responsibility (e.g., see Accenture 2022). Such approaches allow mobilizing available resources in supply chains, similar to Toyota’s supply chain response during the Aisin Seiki fire (Nishiguchi and Beaudet 1998). Ivanov (2021) even suggests that repurposing could be used as an adaptation strategy to maintain supply chain viability during a crisis. While offering important contributions, the supply chain stream fails to capture the complex and interrelated system dynamics that occur between firms, their supply chains, and the external environment during manufacturing repurposing initiatives.

The third notable stream of research in the nascent literature on manufacturing repurposing focuses on innovation. For example, Liu et al. (2021a) explore the effect of shared purpose in driving change in innovation processes and explain how design capability and manufacturing flexibility play key roles in accelerating innovation processes during disruptions. Focusing on the repurposing case of VentilatorChallengeUK, Liu et al. (2021b) highlight open innovation, exaptation,^1^ and ecosystem strategies during the rapid-scale-ups of ventilator production. Poduval et al. (2021) also point out that innovation is one of the main barriers. Relatedly Schwabe et al. (2021) provide a maturity model, which focuses on the speed of innovation diffusion from ideation to market saturation based on the repurposing and customization of existing mass manufacturing infrastructures during the COVID-19 pandemic. Innovation of products, processes, and organizations is key to successful repurposing, but it is not sufficient in its own right.

From the nascent but growing literature on manufacturing repurposing reviewed above, it is clear that it is a multifaceted, complex, and dynamic phenomenon. We aim to bring the facets together in a holistic understanding of the phenomenon. We empirically examine macro-and micro-level interactions within manufacturing repurposing projects, which we use to delineate dynamic cause-and-effect relationships that drive or slow down manufacturing repurposing.

## Research method

**Fig. 1:**
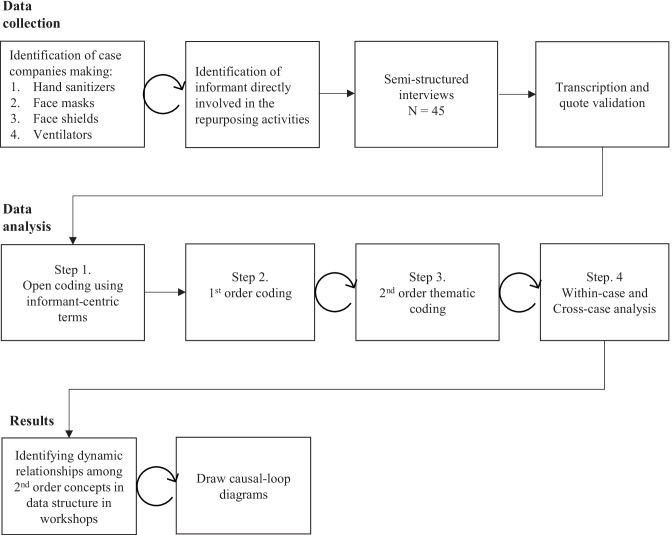
Flowchart of the research process

We set out to build a grounded understanding of manufacturing repurposing. We used an inductive approach based on the systematic collection and analysis of data (Glaser and Strauss 1967; Gioia et al. 2013). To aid in collecting systematic, representative, and in-depth data, we turned to the rich methodological literature on case studies (e.g., Yin 1989; Voss et al. 2002). Case studies summarize insiders’ views of particular events to portray new insights, methods, or techniques. Figure 1 provides a high-level overview of our research process, and the details are explained in the following sections. Curved arrows represent iterations. This section explains the data collection process.

To increase internal validity, we narrowed down the focus of our study to four commonly repurposed products during COVID-19: face shields, face masks, hand sanitizers, and ventilators. The selected healthcare products were among those listed in the World Health Organization’s (WHO) technical guidance on essential resource planning during COVID-19 (WHO 2020). The unit of analysis was manufacturing repurposing operations in factories, including links to the internal and external stakeholders and partners involved, which provided the focal point of the research and served as the basis for sample selection.

To identify respondents, we used purposive and snowball sampling procedures, as explained by Miles and Huberman (1994). We explicitly sought a balanced sample that was not limited to only “successful” repurposing initiatives. The respondents were selected based on their direct involvement in producing these products. First, we focused on the most visible repurposing projects of the selected products in countless media reports and used social media platforms, such as LinkedIn or email, to contact the companies. Second, additional respondents were selected through a snowballing approach in which our primary respondents or contacted persons connected us with another potential respondent. We reached out to around 500 companies, of which about one in ten agreed to participate. In total, we interviewed 45 senior managers from 45 different companies. Semi-structured interviews were conducted from January 2021 to July 2021. The respondents’ profiles are summarized in Table 1.

**Table 1:** Respondents’ profiles

Products	No.	Roles	Experience (Years)	Industries
Face Shields	F1	Project manager	> 10	Consultancy and technological services
	F2	Managing director	> 20	Decontamination solutions
	F3	Consultant	4	Consultancy and technological services
	F4	Head of supply chain	> 20	Manufacturing of sanitary products
	F5	Senior engineer of supply chain	5	Manufacturing of electronics
	F6	Technology director	5	Manufacturing of medical technologies
	F7	Director	> 20	Manufacturing of acrylics
	F8	Director	> 10	Manufacturing of diving equipment
Face Masks	M1	Chief executive officer	> 10	Manufacturing of textiles
	M2	Business development manager	> 20	Manufacturing of textiles
	M3	Sales director	> 20	Manufacturing of textiles
	M4	Chief executive officer	5	Manufacturing of textiles
	M5	Supply chain professional	> 20	Manufacturing of textiles
	M6	Chief executive officer	1	Consultancy and technological services
	M7	Chief executive officer	5	Consultancy and technological services
	M8	Research group head	> 20	Consultancy and technological services
	M9	General manager	> 20	Manufacturing of textiles
	M10	Director of business unit	> 20	Manufacturing of textiles
	M11	Researcher	> 20	Research institution
	M12	Researcher	> 20	Research institution
Sanitizers	S1	Chief executive officer	> 30	Brewery
	S2	Chief executive officer	> 30	Brewery
	S3	Project manager	> 30	Brewery
	S4	Communications manager	> 20	Brewery
	S5	Chief executive officer	3	Distillery
	S6	Project manager	5	Distillery
	S7	Director site logistics/services	> 20	Manufacturing of chemical products
	S8	Project manager	> 10	Manufacturing of chemical products
	S9	Chief executive officer	5	Distillery
	S10	Managing director	> 30	Manufacturing of retail goods
	S11	Chief operations officer	9	Luxury cosmetics
	S12	Site head	8	Pharmaceuticals
Ventilators	V1	Mechanical engineer	> 10	Manufacturing of appliances
	V2	Biomedical engineer	2	Medical products
	V3	Product innovation	> 20	Consultancy and research services
	V4	Managing director	> 20	Machine manufacturer and engineering
	V5	Managing director	> 30	Machine manufacturer and engineering
	V6	Product innovation	> 20	Consultancy and research services
	V7	Researcher	5	Research institution
	V8	Managing director	> 20	Research institution
	V9	Managing director	5	Consultancy and technological devices
	V10	Managing director	5	Research institution
	V11	Researcher	> 20	Research institution
	V12	Project manager	> 10	Research institution
	V13	Researcher	> 10	Research institution

Due to travel restrictions during the pandemic, all interviews were conducted using videoconferencing. The interview questions were separated into two parts: the macro level of the supply chain and external issues and the micro level of firm repurposing operations (the interview guide is included in Appendix A). The semi-structured interviews lasted an average of 65 min. They were recorded, and the relevant content was transcribed. We have collected a qualitative database consisting of 915 pages (185,100 words). The interviews were carried out by two researchers, and the notes were cross-compared after the interviews. Internal reliability was improved by validating the transcribed reports with the informants.

## Analysis

To analyze the data, we used the Gioia method (see Gioia et al. 2013), which is an inductive approach that uses many iterations of analysis to arrive at higher-level concepts. We first carried out open coding with Maxqda software (Berlin, Germany). First- and second-order codes were assigned based on the in-vivo texts from the semi-structured interviews. This thematic coding was then discussed with the research team to reduce coding bias and improve the interpretation of the qualitative data. Second, for our higher-level constructs, we purposefully coded for context, antecedents, enablers, and barriers to manufacturing repurposing. As is common in qualitative research, these steps were iterative. We then conducted a within-case analysis and summarized each case along with the second-order codes. An example is shown in Appendix B, split into the macro level of our analysis (Table B-1 Panel A) and the micro level (Table B-1 Panel B).

Once all cases were coded and described, the next step was a cross-case analysis. We used second-order codes from the interviews to build patterns of key constructs. To structure and present our findings, we applied a data visualization tool from system dynamics called *causal loop diagrams* (see Forrester 1994). This method was selected for its ability to model complex business decisions to form a structural and behavioral representation of the system (Forrester 1961; Sterman 2000). Causal loop diagrams map all essential relationships in a system. They show variables as texts, and the causal relationships among them are represented as arrows. We gradually built the causal loop diagrams through workshops, as we added case after case to the grounded emerging “story.” Consistent with our data, we developed two levels of causal loop diagrams to delineate the relationships between the factors involved externally (macro) and internally (micro) in the firm.

## Results

**Fig. 2:**
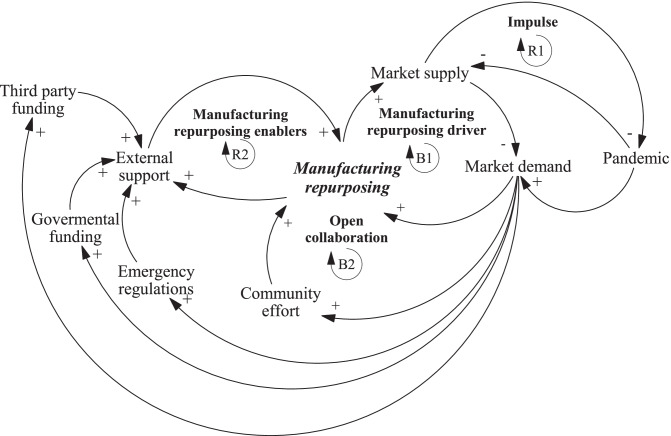
Causal loop diagram of manufacturing repurposing at the macro-level. Notes: *R* Reinforcing loop, *B* Balancing loop

**Fig. 3:**
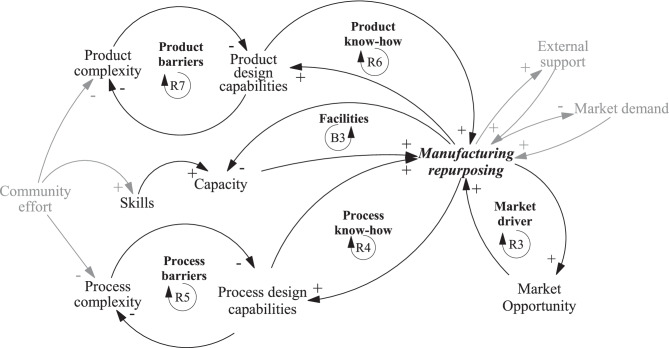
Causal loop diagram of manufacturing repurposing at the micro- level. Notes: *R* Reinforcing loop, *B* Balancing loop

The causal loop diagrams in Figs. 2 and 3 show manufacturing repurposing from the macro-and micro-level perspectives, respectively.

### Macro perspective on manufacturing repurposing

Figure 2 illustrates the system dynamics of manufacturing repurposing at the macro level. The pandemic provides an impulse to increase the market demand for specific products, which is represented by the reinforcing loop R1. Due to the surge in demand, the COVID-19 pandemic led to a supply shortage of PPE and clinical care equipment (CCE) in spring 2020, resulting in a reinforcing loop of supply shortages caused by the pandemic. Thus, a balancing loop was necessary to regulate supply shortages, leading to manufacturing repurposing activities (see balancing loop B1).

Manufacturing repurposing serves as a balancing mechanism for the overall system. It reduces market demand by producing the required PPE and CCE to meet the gaps created by the pandemic. Manufacturing repurposing was seen through various organizations venturing into new product development or collaborating to scale up a legacy product, as denoted by the open collaboration loop (B2). This collaboration played a pivotal role in enabling manufacturing to repurpose supply chains through open-source platforms (i.e., community efforts)*.* Two community effort examples include the WHO’s initiative to publish the formula for manufacturing hand sanitizers online and other firms’ and designers’ initiatives to make 3D printing designs available online for direct printing. This collaborative effort significantly accelerated the manufacturing repurposing process. Manufacturers can bypass the design phase and directly channel their resources into manufacturing and scaling up, resembling the open innovation practices introduced by Chesbrough (2003).

Finally, the reinforcing loop of enablers (R2) supports manufacturing repurposing. Due to the speed and scale at which manufacturing repurposing was required, funding from the government and third parties was imperative. Initiatives such as the VentilatorChallengeUK in the UK, “Ventilators for Canadians,” and “Mechanical Ventilator Milano” are examples of government involvement that supports manufacturing repurposing activities. Furthermore, emergency regulations (e.g., announcing a national “crisis mode” where certification requirements were temporarily lifted for specific products) accelerate the process of manufacturing repurposing by reducing or removing product approval procedures.

### Micro perspective on manufacturing repurposing

The micro-level diagram in Fig. 3 focuses on manufacturing repurposing seen from an individual firm’s perspective. We identified that manufacturing repurposing was reinforced by three critical drivers, represented by market drivers (R3), process know-how (R4), and product know-how (R6), as well as three barriers, represented by facilities (B3), process barriers (R5), and product barriers (R7).

First, the market driver (R3) represents an opportunity for firms to gain either goodwill or business opportunities. It incentivizes organizations to venture into manufacturing repurposing. One of the main drivers for firms to engage in manufacturing repurposing was philanthropy—to help out during times of crisis with no apparent financial motive. Although capturing a new profitable business opportunity was mentioned as the motivation of a few firms, most firms claimed that they engaged in repurposing activities for humanitarian reasons. The role of humanitarian motivation is remarkably different from the usual profit-seeking behavior of companies and hence has important implications for subsequent theory development of manufacturing repurposing.

Second, the process know-how loop (R4) reinforces manufacturing repurposing. Here, existing know-how and project management skills help speed up new product development. For example, it was technologically easy for manufacturers of alcoholic beverages to shift their production capacity to making hand sanitizers. Firms also engaged in agile product development approaches to overcome uncertainty factors in product design requirements. Firms were also seen to apply lean management practices where continuous improvement was carried out to speed up the rate of production. Additionally, we observed that a flat organizational structure with high empowerment for decision making resulted in the effective speed and success of new product development. The speed of new product development was accelerated by applying project management software to monitor various stage-gate processes. The project management software application was seen as an effective tool for coping with the iterations required in response to design-requirement uncertainty. Another effective tool to accelerate processes was 3D printing for rapid prototyping during collaboration. Examples of 3D-printed products include face-shield holders and ventilator parts.

Third, process barriers (R5) represent the challenges that restrain product design capabilities, which impact the repurposing firms’ abilities. In our case studies, process complexities mainly affected the strength of the process barriers. Ventilators were more challenging to produce than the other three products studied, raising the bar for process entry barriers. This higher bar of entry was often overcome by the effect of a community or network approach. Philanthropic acts by both corporate and private entities significantly reduced process barriers by opening access to process designs and capacities. We also observed that firms with established systems and structures for new process establishment and improvement could better manage new product development, manufacturing, and distribution.

Another reinforcing loop concerns product know-how (R6). Product capabilities were critical to the firms as they developed a product that was new to the firm—while simultaneously being under the tremendous pressure of time and resources, as well as experiencing a global pandemic themselves. Consequently, the product know-how was critical to minimizing the number of iterations and errors involved. Overcoming the challenges of high product complexity requires know-how. The challenges were often seen to be reduced through the macro-level loop of community effort that compensates for the lack of internal resources (either tangible or intangible) for the success of repurposing manufacturing.

Next, product barriers (R7) were another roadblock experienced by many case companies. Product complexity reduces the speed of development. Complexities arise due to the nature of the product and the regulatory approvals involved. The complexity of the product is also increased by the number of parts required, along with the sourcing of the parts. During COVID-19, parts’ procurement was challenging, and lead times were long and unpredictable. Like process complexities, product barriers were reduced through community efforts. Capabilities, assets, and experiences were shared across the company and national borders.

Lastly, the facilities loop (B3) serves as a balancing loop for manufacturing repurposing. The need for capacity in the form of physical space, equipment, or resources was imperative due to the organizational changes and scale imposed by the pandemic. In some cases, this capacity was found in-house, and in other cases, the needed capacity was offered by partner firms. Some firms have turned warehouses into new manufacturing spaces for products. The ability to change the setup of current processes and facilities to meet the requirements of the new products also demanded skills and resources. Again, we observed the role of community efforts in contributing to the pool of available skills and resources for the required capacities.

## Discussion

In this section, we discuss the implications of our study for both research and practice. First, our grounded approach led us to identify four theoretical concepts related to manufacturing repurposing, and we discuss these concepts and their implications for future research. Second, for practitioners and policymakers, we explain how this research can improve decision-making processes in preparation for and during future emergencies.

### Implications for research

From our analysis, four theoretical concepts appear particularly relevant during manufacturing repurposing initiatives: (1) open innovation, (2) dynamic capabilities, (3) agile product development, and (4) corporate philanthropy in manufacturing repurposing. Below, we discuss how future research can advance manufacturing repurposing research by building on these concepts.

#### Open innovation

We observed a significant level of collaboration across the firm’s boundaries during manufacturing repurposing from our empirical results. For firms to successfully engage in manufacturing repurposing, capabilities (e.g., production know-how) or capacities were sourced beyond the firms’ boundaries. Sourcing beyond the firms’ boundaries was evident to a certain extent across all case companies, including different types and levels of collaboration widespread across the entire value chain processes. For instance, in one company, collaboration with another organization facilitated the development process of the ventilator. We also observed know-how sourcing from online communities in several of the case companies. Theoretically, this type of beyond-company-border information search and collaboration has been called “open innovation” (Chesbrough 2006). The application of open innovation accelerated the manufacturing repurposing activities of the firms. From an operations management perspective, we suggest further research on how firms can manage, implement, and assimilate external ideas into their operations during emergencies. A related and interesting question addresses the evolution of the newly established collaborations when crises pass. Researchers could also study the extent to which companies can benefit from open innovation practices to increase their preparedness for future manufacturing repurposing.

#### Dynamic capabilities

We found that companies benefited from product-and process-related routines. In particular, innovating quickly and implementing new product and process designs are essential during manufacturing repurposing initiatives. This ability has been called “dynamic capabilities” (Teece et al. 1997; Eisenhardt and Martin 2000). Dynamic capabilities are “the capacity to renew competences so as to achieve congruence with the changing business environment” by “appropriately adapting, integrating, and reconfiguring internal and external organizational skills, resources, and functional competencies to match the requirements of a changing environment” (i.e., capabilities) (Teece et al. 1997, p. 515). The fit of this theory to manufacturing repurposing has already been demonstrated by Ramos et al. (2021) and Puliga and Ponta (2021). Future research could further explore the dynamic capabilities that companies can develop prior to emergencies, which can potentially be evoked and help improve manufacturing repurposing operations.

#### Agile product development

Our empirical evidence repeatedly points to the application of *agile* product/hardware development. Agile product development involves a quick method of product development in which sub-solutions are developed and tested iteratively with the customer (Takeuchi and Nonaka 1986). Agile product development revolves around six characteristics: built-in instability, self-organizing project teams, overlapping development phases, multi-learning, subtle control, and organizational transfer of learning (Takeuchi and Nonaka 1986). This concept was further advanced by Schwaber (1997) into the SCRUM^2^ framework as a process for software product release. While agile was historically developed for *software* products, agile *hardware* development is a practical approach for rapidly creating physical systems with high potential for manufacturing repurposing (Omidvarkarjan et al. 2020). For instance, one case company followed an agile product development approach to respond quickly to the ever-changing regulations and requirements of the product. Future research can build on early work that studies manufacturing repurposing using the concept of agile product development. For example, Schmidtner et al. (2021) discuss the role of agile working during the pandemic and its impact on the future of work environments; Janssen and Van der Voort (2020) explain the complementary (and sometimes contradictory) role of agility and adaptivity governance during the COVID-19 pandemic; and more broadly, Yayla-Küllü et al. (2021) advocate that firms need to increase the extent of their agile and adaptable operations (e.g., by using product-line flexibility or in general resource flexibility) to cope with uncertainties.

#### Corporate philanthropy

One of the most striking features of the manufacturing repurposing initiatives was that organizations did not repurpose just for the sake of economic rent. Most of the ad hoc initiatives of firms were driven by goodwill to help healthcare workforces and societies combat the shortages of critical items. The initiatives were almost always driven by individuals passionate about and driven to help out during COVID-19—sometimes as a response to a request from local governments. This important aspect of manufacturing repurposing can be studied using the notion of corporate philanthropy. Corporate (strategic) philanthropy is defined “as the synergistic use of organizational core competencies and resources to address key stakeholders’ interests and to achieve both organizational and social benefits” (McAlister and Ferrell 2002, p. 690). The notion of corporate philanthropy offers several promising research paths. For example, how can we study manufacturing repurposing from the perspective of corporate philanthropy? How are firms’ philanthropic decision-making processes affected by firm antecedents and ownership structures (e.g., private vs. public, small vs. large, etc.)? What is the role of operations managers in philanthropic activities? How will the experiences from manufacturing repurposing during the COVID-19 pandemic impact firms’ willingness to engage in philanthropic activities in the future?

### Implications for practice

Our findings offer advice for manufacturing managers and policymakers. Overall, for both interest groups, we provide a visual overview of the most central driving and braking forces of manufacturing repurposing—both at the macro and micro perspectives. The causal loop diagrams help inform decisions because they show the likely effects of interventions.

For manufacturing managers, our results highlight, in particular, the importance of collaboration within and between firms. Success in repurposing initiatives depends largely on the network. Through collaborations (e.g., buyer–supplier relationships), firms can mobilize a larger pool of resources, which provides better access to product and process know-how and designs. Maintaining a close relationship with key partners and suppliers provides access to the required know-how and capabilities. If these collaborative relationships are built before a crisis, they are much easier and quicker to evoke when needed. The more complex the product, the more important the role of the network is. Among the products that we studied, within-and between-firm collaborations were essential for ventilator production and less evident for sanitizers. Relatedly, firms with agile internal product and process design capabilities exhibited more successful manufacturing repurposing operations. They rallied more people faster to the repurposing mission and drew on the necessary internal and external expertise and information to design the products and processes. Again, this capability can be nurtured and built before the next crisis.

For policymakers, we first point out anecdotal evidence that firms that collaborate closely with the agencies responsible for developing emergency regulations report a higher success rate in their manufacturing repurposing projects. Second, we emphasize the role of policymakers in general. During a crisis, companies should not be left alone. While market forces help create economic incentives to engage in manufacturing repurposing, companies need assistance in building collaborative networks, receiving funding, and navigating juridical frameworks. Furthermore, because corporate philanthropy plays a large role, governments should not rely on market forces to make repurposing happen. Closer relationships and frequent and faster feedback cycles between regulatory agencies and manufacturers generally result in faster product development processes with fewer iterations and more effective products for end users. Therefore, a high degree of collaboration between firms, governments, and regulatory agencies increases the success of manufacturing repurposing. Such collaborative frameworks should be developed before or during the early stages of emergencies.

### Limitations and outlook

Our study has several limitations. Most importantly, although our research is based on the largest multiple-case study of manufacturing repurposing in the literature, we are limited by having only qualitative insights. Future research could provide quantitative evidence. For example, one promising research direction is to map the continuum of case companies from unsuccessful to successful manufacturing repurposing projects—in terms of, for example, the degree to which they manufactured the intended products at scale—and reflect on the characteristics, hurdles, and best practice. For example, is there a link between companies with established lean practices or flexible manufacturing operations and their success or unsuccess rates during manufacturing repurposing? Scholars drawing on quantitative evidence can build maturity models to assess the best practices and readiness levels of firms and societies to engage in manufacturing repurposing projects. Future quantitative research could also explore the role of contextual factors, such as the size of companies, product complexity, technological capabilities, organizational culture, and country characteristics in manufacturing repurposing projects.

There is also a range of other potentially relevant and interesting research streams. Researchers can study how companies design, prototype, and develop radically different products and what they can learn from their experiences. Future scholars can also examine the regulatory aspects of manufacturing repurposing, such as contracts, intellectual property, standardizing processes, and licensing. The open-source culture movement, including both software and hardware, can be studied. Other promising research avenues are the role of new technologies, mainly additive manufacturing, digital platforms, digital infrastructure, and advanced robots. Future research can also study organizational-related aspects, such as project management, team building, leadership, knowledge management, organizational learning, training, and organizational design during manufacturing repurposing projects. In short, manufacturing repurposing offers a broad pallet of relevant and interesting research avenues for scholars.

## Conclusion

This research has provided an in-depth understanding of manufacturing repurposing, drawing on rich empirical insights from the manufacturing repurposing of four product categories during the early stages of the COVID-19 pandemic: face shields, face masks, hand sanitizers, and ventilators. To structure our analysis, we systematically coded the interview data to identify the key constructs of the manufacturing repurposing phenomenon. We summarized the findings in two causal loop diagrams at the macro and micro levels. These diagrams visualize and conceptually illustrate the dynamic behavior of manufacturing repurposing operations. Our study contributes one of the first empirical analyses of the manufacturing repurposing phenomenon to the literature.

## Appendix A. Interview guide

### Part 1: Impact of Covid-19

1. What was the impact/challenges of Covid-19 on your factory?
2. What were the operational changes to your production lines/processes due to Covid-19? (if any)

### Part 2: Repurposing manufacturing *(Micro Level)*

3. How did your factory move from producing core products to the new repurposed products?
  - Installing new machines/equipment, using new technologies, and setting up new production lines?
  - Training staff?
  - Defining new organizational practices?
  - How long did it take for you to implement changes?
4. What were the main challenges?
  - Macro Level: Regulatory, funding, competition, distribution
  - Firm Level: Know-how, manpower, facilities
5. What was the main driving force/motivation to repurpose and who made the decision to repurpose?
  - Top management
  - Team wanted to make a change
  - Goodwill/ Mandatory obligations
6. Are still producing that product? If not:
  - Why did you stop?
  - What have you done with the equipment? Will you dispose of them, store them, sell them?
  - What have you done with the excess capacity?
7. What were the best practices/learnings from repurposing?
  - How to cope with uncertainty
  - New organizational practices
  - New ways of managing operations/new project management style
  - Knowledge management of competencies/ know-how

### Part 3: Repurposing manufacturing *(Macro Level)*

8. How did you manage the disruption from the suppliers’ side?
9. How did you distribute your products to the customers? How did you identify them? Were there any challenges?
10. How could you improve the supply chain of manufacturing repurposing for the future?
  - How did this change your current organizational management of supply chains (e.g., multiple suppliers, digital systems)
  - Suppliers’ relationships

## Appendix B. Coding Approach

### Table B-1 Excerpts of Coding

**Table:**

**Panel A.** Macro Level			
**Examples of In-Vivo Coding**	**Source**	**1st-Order Code**	**2nd-Order Code**
“It was a call from the government in which manufacturers were asked to help in any way possible.”	V1	Motivated by the government	External support
“It was a response to the UK government as we were contacted.”	V6		
“We got a message from the council to help in any way or form that we could.”	V8		
“We were provided with the resources and funding that were required by the government.”	V3	Funding by the government	
“There was an accelerated pathway for regulations, and thus this sped up the process.”	V10	Emergency regulations	
“Regulations were constantly changing, but the emergency requirements helped it go faster.”	F8		
“A reduced sentence was agreed upon by the MHRA [Medicines and Healthcare products Regulatory Agency] for approval.”	V12		
“The difference is that the regulator worked with us in parallel to understand how…to trim back the regulations without compromising the safety of the patient.”	V8	Early involvement of regulators	
“What was driving us was mainly our calls with the clinicians, as they were mainly the people in the frontline.”	V4		
“I was driving there myself, and it is important to build relationship—to show that you are willing and doing it properly and doing it for the country.”	S2	Good relationship with the supplier	Community effort
“There was also tremendous support from the support services, such as DHL, accountants and lawyers, to draft the necessary contracts required.”	V4	Strategic partnerships and influence of organization	
“I was not doing it for the job title or the paycheck; I was trying to help in any way that I could.”	V1	Motivation to help the community	
“Something is happening in our community, and we wanted to support it in whatever way, however possible.”	V3		
“We were working around the clock and utilizing the different time zones to work 24 h.”	V7		
“It was mainly to help wherever possible given the dire pandemic situation at that time.”	V11		
“There was a massive rescheduling required to meet the demands in such a short time.”	F7	Managing demand in manufacturing	Market demand
“The global supply dried up because you couldn’t really manufacture fast enough to meet the demands.”	M3		
“We scaled down R&D and new product development to make time for face shields.”	F2	Prioritizing production schedule	
“We were able to schedule this order during our regular four-shift production.”	F5	Managing shifts to meet demand	

**Table:**

**Panel B.** Micro Level			
**Examples of In-Vivo Coding**	**Source**	**1st-Order Code**	**2nd-Order Code**
“It is a new product because we are not a medical device company.”	V1	Challenges in product development	Product design capabilities
“I had experience in mechanical engineering and thus contributed to the design and development aspect as well as the quality and inspection side.”	V3		
“Clear constraints and limitations of the product should be identified prior to the design of the ventilator.”	V9	Coordination and visibility in times of chaos	Process design capabilities
“It was difficult because there was no pathway. There was no clear leadership available to guide us through.”	F1		
“We just changed lanes and did what we usually do in a different lane.”	V6		
“We asked them how they would design their production lines and used their expertise for that.”	V7	Manufacturing process skills	
“We repurposed our skills to do something else but of a much higher complexity.”	V6	Know-how applied to new products	Product complexities
“There wasn’t much engineering behind this. I would say it is more of an arts and crafts project.”	F5		
“From the process viewpoint, it was similar to previous projects, as we were used to the development and innovation of medical devices.”	V8	Organizational processes	Process complexities
“We are using the processes that we are familiar with but at a larger scale.”	V10		
“Aerospace and automotive industries may find it less different as they have QMS [quality management system] processes related to safety and are similar to medical requirements.”	V4	Quality processes	
“We applied the ERP system, which helped us to keep an eye on the raw materials during the times of crisis.”	V5	Application of digital technologies for processes	

## Footnotes

1 The “ability to ‘pivot’ … from one function to another, without the need for a long and costly development process” (Liu et al. 2021a, p. 2).

2 SCRUM is a “management, enhancement and maintenance methodology for an existing system or production prototype” coined after the term in rugby – a tight formation of forwards who bind together in specific positions when a scrumdown is called (Schwaber 1997, p. 118).
